# Physical Activity Dose and Depression in a Cohort of Older Adults in The Irish Longitudinal Study on Ageing

**DOI:** 10.1001/jamanetworkopen.2023.22489

**Published:** 2023-07-10

**Authors:** Eamon Laird, Charlotte Lund Rasmussen, Rose Anne Kenny, Matthew P. Herring

**Affiliations:** 1 Physical Activity for Health Research Centre, Health Research Institute, University of Limerick, Limerick, Ireland; 2 The Irish Longitudinal Study on Ageing, Trinity College Dublin, Dublin, Ireland; 3 Department of Physical Education and Sport Sciences, University of Limerick, Limerick, Ireland; 4 Curtin School of Allied Health, Faculty of Health Sciences, Curtin University, Perth, Australia

## Abstract

**Importance:**

Among older adults (aged ≥50 years), depression is associated with an increased risk of physical, social, and cognitive dysfunction. Regular moderate to vigorous physical activity (MVPA) has been associated with lower odds of depression. However, the lowest dose for protection against depression and the extent to which exceeding this level conveys additional protection are unknown.

**Objective:**

To evaluate different MVPA doses, depressive symptoms, and major depression status in a large cohort of older adults with and without chronic disease.

**Design, Setting, and Participants:**

A longitudinal cohort study of the same 4016 individuals at each of 5 time points (ie, waves) from The Irish Longitudinal Study on Ageing was conducted. Data were collected from October 2009 to December 2018, and data were analyzed from June 15 to August 8, 2022.

**Exposures:**

Continuous MVPA (metabolic equivalent of task [MET]–minutes per week [MET-min/wk]), 3 dose categories, and 5 dose categories measured with the International Physical Activity Questionnaire.

**Main Outcomes and Measures:**

Depressive symptoms and major depression status were measured using the short form of the Centre for Epidemiological Studies Depression scale along with the Composite International Diagnostic Interview for diagnosis of a major depressive episode during the past 12 months. Multivariable negative random-effects binomial regression models, adjusted for relevant covariates, quantified associations across time.

**Results:**

Among the 4016 participants at each wave of the study (2205 women [54.9%]; mean [SD] age, 61.0 [8.1] years) during 10.0 years of follow-up, depression rates increased from a mean of 8.2% (95% CI, 7.4%-9.1%) to 12.2% (95% CI, 11.2%-13.2%). Bonferroni-corrected post hoc analysis indicated that participants performing 400 to less than 600 MET-min/wk had a 16% lower rate of depressive symptoms (adjusted incidence rate ratio [AIRR], 0.84; 95% CI, 0.81-0.86) and 43% lower odds of depression (adjusted odds ratio [AOR], 0.57; 95% CI, 0.49-0.66) compared with 0 MET-min/wk. Those with chronic disease performing 600 to less than 1200 MET-min/wk had an 8% (AIRR, 0.92; 95% CI, 0.86-0.98) lower rate of depressive symptoms and 44% (AOR, 0.56; 95% CI, 0.42-0.74) lower odds of depression compared with 0 MET-min/wk. Those without disease required more than 2400 MET-min/wk for similar protection for depressive symptoms (AIRR, 0.81; 95% CI, 0.73-0.90).

**Conclusions and relevance:**

In this cohort study of older adults, significant antidepressant benefits were noted for MVPA doses below current recommendations for overall health, although greater doses were associated with larger AIRR reductions. It may be useful for public health interventions to investigate the achievability of lower physical activity thresholds among older adults with and without chronic illness to reduce the risk of depression.

## Key Points

**Question:**

What is the minimal dose of moderate to vigorous physical activity (MVPA) associated with a reduced risk of depression and depressive symptoms in older adults (aged ≥50 years) with and without chronic disease?

**Findings:**

In this cohort study among 4016 older adults, at each of the included time points across a 10-year period, a negative dose-response association was observed between MVPA dose and depressive symptoms and major depression.

**Meaning:**

The findings of this study suggest that a lower dose of MVPA than recommended from guidelines for overall health may be associated with a lower risk of depression among older adults.

## Teaser

This cohort study examines depressive symptoms and major depression in older adults with and without chronic disease performing varying levels of physical activity over a 10-year period.

## Introduction

Depressive symptoms and disorders are prevalent and debilitating among older adults, being substantial risk factors for major chronic conditions, including cognitive and functional decline, cardiovascular disease and chronic pain, and increased risk of all-cause mortality and suicide.^1,2,3,4^ Depression causes more than 5% to 10% of the burden of all diseases in Europe relative to disability-adjusted life years,^5^ and the economic burden in the US alone is estimated to be more than $210.5 billion.^6^ Identification of potentially accessible and low-cost health and lifestyle behaviors that could attenuate risk factors for depressive symptoms and disorders, particularly among those with chronic illnesses, remains a high priority. Recent meta-analytic evidence^7^ and previous prospective cohort findings^8^ support the prophylactic effects of regular physical activity for depression; across 111 prospective cohort studies including more than 3 million adults, after physical activity exposure the odds of incident cases of depression or an increase in subclinical depressive symptoms were reduced by 21% in fully adjusted models.^7^ This review highlighted critical knowledge gaps, including a paucity of evidence on the minimal effective physical activity dose for depression and the physical activity dose associated with the greatest reduction in odds of depression over time. This is further supported by reviews by the World Health Organization (WHO) Guideline Development Group,^9^ which reported that knowledge on physical activity and the mental health dose-response, especially optimal dose, from adequately powered studies among individuals with chronic disease has remained limited. Randomized clinical trials have shown that depressive symptom reductions following exercise training are significantly larger among patients with chronic illness who were meeting recommended physical activity levels.^10^ However, to our knowledge, there is no consensus on how much physical activity is protective for depression overall or whether this may vary among those with and without chronic disease.

Thus, we aimed to quantify the lowest dose of moderate to vigorous physical activity (MVPA) associated with reduced odds of depressive symptoms and major depression status among older adults with and without chronic disease across 10 years, using data from waves 1 to 5 of The Irish Longitudinal Study on Ageing (TILDA). A secondary aim was to examine the importance of meeting and exceeding the identified minimally sufficient dose compared with current physical activity recommendations to protect against depression with the traditional WHO 3 dose categories^9^ and recently suggested 5 dose categories.^7^

## Methods

### Study Design and Population

TILDA is a population representative study of older adults (aged ≥50 years) residing in the Republic of Ireland. Previous details of TILDA survey design, participant sampling, and data collection methods have been published.^4^ This research used 10 years of TILDA data (waves 1-5; n = 8507) (October 2009-December 2018), with each data collection wave containing the same participants followed up across time. The data collection at each wave included detailed information on demographic, health, lifestyle, and social factors through either a self-completed questionnaire, nurse health assessment, or interview. At wave 1 (2009-2011) and wave 3 (2014-2015), each participant completed a nurse health assessment. At wave 2 (2012-2013), wave 4 (2016), and wave 5 (2018), participants completed interviews and questionnaires. We excluded those with missing data on depression or physical activity at each wave, in addition to those who reported walking or MVPA greater than a combined 16 hours per day, died, or dropped out of the study (n = 4491), leaving a final analytical sample of 4016 participants at each wave with all variables of interest. This longitudinal cohort study was approved by the Faculty of Health Sciences Research Ethics Committee at Trinity College Dublin, and all participants gave informed written consent; participants did not receive financial compensation. All experimental procedures adhered to the Declaration of Helsinki,^11^ and all assessments were performed by trained research nurses. This study followed the reporting requirements of the Strengthening the Reporting of Observational Studies in Epidemiology (STROBE) reporting guideline.

### Ascertainment of Outcome

At all waves, depressive symptoms were measured using the short form of the Centre for Epidemiological Studies Depression (CES-D); the continuous sum score was used to quantify depressive symptoms and for analysis of depressive symptoms. This shortened 8-item version of the CES-D has been validated against the 20-item scale within the TILDA cohort, and the latent factors of depression from the short-form version have been shown to be consistent, reliable, and valid in community-living older adults.^4^ Additionally, from waves 2 to 5, the Composite International Diagnostic Interview quantified diagnosis of a major depressive episode during the past 12 months. Major depression status was classified as either a CES-D score greater than or equal to 9 at any wave and/or a major depressive episode at any wave.

### Assessment of Physical Activity

Across all data collection waves, self-reported physical activity was measured using the short-form International Physical Activity Questionnaire. Participants were asked to report the total number of days and duration of vigorous, moderate, and walking activities during the previous 7 days. Data were summed to estimate the total number of metabolic equivalent of task (MET) minutes (defined as the amount of oxygen consumed while sitting at rest) engaged in weekly and categorized into MVPA doses (MET-min/wk). The MVPA dose was quantified in 3 ways: (1) traditional 3 dose categories: low (<600 MET-min/wk), moderate (600 to <1200 MET-min/wk), or high (≥1200 MET-min/wk)^8^; (2) expanded 5 dose categories (0, 1 to <600, 600 to <1200, 1200 to <2400, and ≥2400 MET-min/wk)^7^; and (3) minimally sufficient continuous physical activity dose (METs per minute per week) (the minimal number of METs per minute per week needed to observe an association with the outcome measure).

### Covariates

Covariates included age (continuous), sex (female and male), educational level (primary, secondary, or tertiary), and antidepressant use (yes or no). The population was predominantly White at the initial recruitment date; therefore, this question was not asked. Self-reported health information was examined for chronic disease burden, with respondents asked specifically about a history of chronic diseases that included cardiovascular disease (prior myocardial infarction, cardiac failure, angina, hypertension, or cardiac arrhythmia), lung disease, osteoporosis, cancer, liver disease, age-related macular degeneration, cataracts, glaucoma, arthritis, urinary incontinence, Parkinson disease, and diabetes. This was then classified as chronic disease category (with or without). Lifestyle factors included smoking status (current or past/never) and excessive alcohol use through the Cut down, Annoyed, Guilty, Eye-opener questionnaire with a score of 2 or greater indicating excessive alcohol use (yes or no). Body mass index was quantified as Quetelet Index using nurse-measured height and weight at health collection waves or self-reported at other data collection waves (obesity defined as body mass index ≥30, calculated as weight in kilograms divided by height in meters squared).

### Statistical Analysis

Statistical analyses were performed from June 15 to August 8, 2022, and used depression and physical activity at all time points. The continuous sum score of the CES-D was used in analysis of depressive symptoms; however, overdispersion of these data precluded the use of Poisson regression models. Thus, several models of mixed-effects regression with a negative binomial link function quantified associations between each MVPA dose measurement: 3 dose physical activity (model 1), 5 dose physical activity (model 2), and continuous METs per minute per week (model 3) and depressive symptoms. Model 4a and model 4b were adjusted for wave (time panel), age, and sex; models 5a and 5b were further adjusted for factors known to be associated with depression, including educational level, location, current smoking and alcohol use, and antidepressant medication use. Random effects for each participant were included in all models to allow for correlation between observations at each time point. For major depression status, several models of mixed-effects regression with a logistic link function quantified associations of both 3 dose (model 4) and 5 dose (model 5) physical activity with major depression status. Model 4a and model 4b were adjusted for wave (time panel), age and sex; models 5a and 5b were further adjusted for factors known to be associated with depression, including educational level, location, current smoking and alcohol and use, antidepressant medication use.

A Bonferroni-corrected post hoc analysis was conducted to quantify associations between lower MVPA doses within the 1 to less than 600 MET-min/wk category and depressive symptoms and major depression status by repeating the primary analysis with a new 4-dose physical activity variable consisting of 0, 1 to less than 200, 200 to less than 400, and 400 to less than 600 MET-min/wk. Furthermore, 2 sensitivity analyses were conducted by further adjusting all models for obesity and removing participants performing 0 MET-min/wk. Analyses were repeated for all models after stratifying by the presence or absence of chronic disease (ie, those with and without chronic illness). Adjusted incidence rate ratios (AIRRs) and adjusted odds ratios (AORs) are reported, and statistical analyses were conducted using Stata, version 14.1 (StataCorp LLC). For baseline comparisons only, a 2-sided, paired test was used, with *P* < .05 set as the threshold for significance.

## Results

Among the 4016 participants at each wave with depression and physical activity measures in the study (20 080 responses in total; 2205 women [54.9%]; 1811 men [45.1%]; and mean [SD] age, 61.0 [8.1] years) during 10.0 years of follow-up, depression rates increased from a mean of 8.2% (95% CI, 7.4%-9.1%) to 12.2% (95% CI, 11.2%-13.2%) (eFigure in Supplement 1). During this period, prescribed antidepressant medication use increased from 6.1% (95% CI, 5.4%-6.9%) to 10.3% (95% CI, 9.3%-11.2%). Full demographic characteristics of the cohort are presented in Table 1. During the 10-year period, the proportion of those meeting or exceeding physical activity guidelines (>600 MET-min/wk) decreased from 51.1% (95% CI, 49.5%-52.6%) at wave 1 to 40.9% (95% CI, 39.3%-42.4%) at wave 5.

**Table 1.** Baseline Characteristics of Participants From Time Panel Data Across Waves 1 to 5 (2009-2018)

Variable	Panel data collection (N = 4016 in each wave), No. (%)				
	Wave 1	Wave 2	Wave 3	Wave 4	Wave 5
Age, mean (SD), y	61.7 (8.1)	63.7 (8.1)	66.1 (8.2)	68.2 (8.2)	70.2 (8.1)
Sex					
Female	2205 (54.9)	2205 (54.9)	2205 (54.9)	2205 (54.9)	2205 (54.9)
Male	1811 (45.1)	1811 (45.1)	1811 (45.1)	1811 (45.1)	1811 (45.1)
Educational level					
Primary	876 (21.8)	878 (21.9)	876 (21.8)	870 (21.7)	867 (21.6)
Secondary	1669 (41.6)	1608 (40.0)	1585 (39.5)	1579 (39.3)	1568 (39.0)
Tertiary	1470 (36.6)	1529 (38.1)	1555 (38.7)	1567 (39.0)	1581 (39.4)
Lifestyle factors					
Current smoker	600 (15.0)	526 (13.1)	453 (11.3)	414 (10.3)	393 (9.8)
Excess alcohol use^a^	470 (13.0)	462 (13.3)	434 (12.5)	417 (11.8)	393 (9.8)
Obesity^b^	1023 (33.0)	NA^c^	860 (21.4)	895 (22.3)	854 (21.3)
Antidepressant use	246 (6.1)	312 (7.8)	350 (8.7)	357 (8.9)	412 (10.3)
Chronic disease					
With	3086 (77.1)	3145 (79.1)	3258 (81.2)	3318 (82.8)	3333 (83.0)
Without	915 (22.9)	833 (20.9)	755 (18.8)	690 (17.2)	683 (17.0)
MVPA, MET-min/wk					
3 Dose category					
Low (0 to <600)	1965 (48.9)	1919 (47.8)	2284 (56.9)	2239 (55.8)	2374 (59.1)
Moderate (600 to <1200)	279 (6.9)	352 (8.8)	303 (7.5)	318 (7.9)	372 (9.3)
High (≥1200)	1722 (44.2)	1745 (43.5)	1429 (35.6)	1459 (36.3)	1270 (31.6)
5 Dose category					
1 (0)	1591 (39.6)	1476 (36.8)	1868 (46.5)	1793 (44.6)	1915 (47.7)
2 (1 to <600)	374 (9.3)	443 (11.0)	416 (10.4)	446 (11.1)	459 (11.4)
3 (600 to <1200)	279 (6.9)	352 (8.8)	303 (7.5)	318 (7.9)	372 (9.3)
4 (1200 to <2400)	518 (12.9)	481 (12.0)	433 (10.8)	413 (10.3)	400 (10.0)
5 (≥2400)	1254 (31.2)	1264 (31.5)	996 (24.8)	1046 (26.0)	870 (21.7)

Abbreviations: MET, metabolic equivalent task; MVPA, moderate to vigorous physical activity; NA, not applicable.

^a^ Excess alcohol use defined as a score of 2 or greater on the Cut down, Annoyed, Guilty, Eye-opener questionnaire.

^b^ Defined as body mass index of 30 or greater (calculated as weight in kilograms divided by height in meters squared). In total, 3966 participants had obesity measures due to missing data.

^c^ Information not collected at wave 2.

### Analysis of MVPA Doses and Depressive Symptoms in Overall Sample

Results of the analysis of MVPA doses and depressive symptoms are reported in Table 2. No association between continuous MET minutes per week and depressive symptoms was observed after investigating the potential for any curvilinear association using linear and quadratic terms for physical activity. A dose-response association was observed between MVPA dose categories and depressive symptoms. For the 3 dose categories, the rates of depressive symptoms were 20% lower among individuals with high physical activity compared with individuals with low physical activity (AIRR, 0.80; 95% CI, 0.78-0.84). The results of the 5 dose MVPA categories revealed an incremental decrease in depressive symptoms for each increase in physical activity dose, with 7% (AIRR, 0.93; 95% CI, 0.88-0.99), 16% (AIRR, 0.84; 95% CI, 0.80-0.89), and 23% (AIRR, 0.77; 95% CI, 0.74-0.80) lower rates of depressive symptoms among those meeting recommendations (600 to <1200 MET-min/wk), exceeding recommendations (1200 to <2400 MET-min/wk), and with the highest doses (≥2400 MET-min/wk) compared with those performing 0 MET-min/wk. Bonferroni-corrected post hoc analysis (Table 2) showed that participants performing 400 to less than 600 MET-min/wk had a 16% lower rate of depressive symptoms compared with participants performing 0 MET-min/wk (AIRR, 0.84; 95% CI, 0.81-0.86).

**Table 2.** AIRRs for Associations of MVPA With Depressive Symptoms and AORs for Associations With Depression Status

PA dose type (MET∙min∙wk^−1^)	Depressive symptoms		Major depression status	
	AIRR (95% CI)^a^	AIRR (95% CI)^b^	AOR (95% CI)^a^	AOR (95% CI)^b^
Continuous	1.00 (1.00-1.00)	1.00 (1.00-1.00)	1.00 (1.00-1.00)	1.00 (1.00-1.00)
3 Dose PA				
Low	1 [Reference]	1 [Reference]	1 [Reference]	1 [Reference]
Moderate	0.93 (0.88-0.97)	0.95 (0.90-1.00)	0.53 (0.41-0.67)	0.58 (0.45-0.75)
High	0.80 (0.77-0.82)	0.80 (0.78-0.84)	0.50 (0.43-0.57)	0.56 (0.48-0.65)
5 Dose PA				
0	1 [Reference]	1 [Reference]	1 [Reference]	1 [Reference]
1 to <600	0.92 (0.88-0.97)	0.93 (0.89-0.99)	0.74 (0.61-0.90)	0.86 (0.69-1.05)
600 to <1200	0.91 (0.86-0.96)	0.93 (0.88-0.99)	0.49 (0.39-0.62)	0.56 (0.43-0.73)
1200 to <2400	0.84 (0.79-0.88)	0.84 (0.80-0.89)	0.52 (0.42-0.64)	0.59 (0.47-0.74)
≥2400	0.76 (0.73-0.79)	0.77 (0.74-0.80)	0.44 (0.37-0.52)	0.51 (0.43-0.62)
Post hoc refined low-dose PA^c^				
0	1 [Reference]	1 [Reference]	1 [Reference]	1 [Reference]
1 to <200	0.92 (0.84-1.01)	0.94 (0.85-1.04)	0.70 (0.47-1.05)	0.82 (0.53-1.26)
200 to <400	0.95 (0.87-1.01)	0.95 (0.88-1.02)	0.78 (0.59-1.05)	0.91 (0.67-1.24)
400 to <600	0.82 (0.79-0.84)	0.84 (0.81-0.86)	0.50 (0.43-0.56)	0.57 (0.49-0.66)

Abbreviations: AOR, adjusted odds ratio; AIRR, adjusted incidence rate ratio; MET, metabolic equivalent task; MVPA, moderate to vigorous physical activity; PA, physical activity.

^a^ Model adjusted for age (continuous), sex (reference, male), and wave (reference, baseline).

^b^ Model adjusted for age (continuous), sex (reference, male), wave (reference, baseline), educational level (reference, primary), smoking status (reference, nonsmoker), excess alcohol use (reference, none), and antidepressant medication use (reference, nonuse).

^c^ Analysis for refined low-dose PA based on the population reporting less than 600 MET-min/wk, with 10 791 participants adjusted for age (continuous), sex (reference, male), and wave (reference, baseline), and 9207 participants fully adjusted to previous variables in addition to educational level (reference, primary), smoking status (reference, nonsmoker), excess alcohol use (reference, none), and antidepressant medication use (reference, nonuse).

### MVPA Dose and Depressive Symptoms Among Those With and Without Chronic Disease

For the 3 dose categories, compared with those in the low-dose category, those in the high-dose category with chronic disease had 21% (AIRR, 0.79; 95% CI, 0.76-0.83) lower rates of depressive symptoms, and those without chronic disease had 10% (AIRR, 0.90; 95% CI, 0.82-0.99) lower rates of depressive symptoms (Table 3). For the 5 dose categories, those with chronic disease showed reduced rates of depression symptoms beginning at 600 to less than 1200 MET-min/wk (AIRR, 0.92; 95% CI, 0.86-0.98), whereas those without chronic disease showed reduced rates only beginning at greater than 2400 MET-min/wk (AIRR, 0.81; 95% CI, 0.73-0.90) (Table 3). Regardless of disease status, performing 2400 MET-min/wk or more was associated with the greatest risk reduction; no association was found for continuous MET minutes per week.

**Table 3.** AIRRs for Associations of MVPA With Depressive Symptoms in Those With and Without Chronic Disease

PA dose type (MET∙min∙wk^−1^)	With disease (n = 16 204)		Without disease (n = 3490)	
	AIRR (95% CI)^a^	AIRR (95% CI)^b^	AIRR (95% CI)^a^	AIRR (95% CI)^b^
Continuous	1.00 (1.00-1.00)	1.00 (1.00-1.00)	1.00 (1.00-1.00)	1.00 (1.00-1.00)
3 Dose PA				
Low	1 [Reference]	1 [Reference]	1 [Reference]	1 [Reference]
Moderate	0.91 (0.86-0.96)	0.93 (0.88-0.99)	0.98 (0.86-1.13)	1.03 (0.89-1.19)
High	0.79 (0.76-0.82)	0.79 (0.76-0.83)	0.85 (0.78-0.93)	0.90 (0.82-0.99)
5 Dose PA				
0	1 [Reference]	1 [Reference]	1 [Reference]	1 [Reference]
1 to <600	0.94 (0.89-0.98)	0.96 (0.91-1.01)	0.84 (0.74-0.96)	0.88 (0.76-1.01)
600 to <1200	0.90 (0.85-0.95)	0.92 (0.86-0.98)	0.94 (0.82-1.09)	0.99 (0.85-1.15)
1200 to <2400	0.81 (0.77-0.86)	0.82 (0.77-0.87)	0.95 (0.84-1.07)	1.00 (0.88-1.14)
≥2400	0.76 (0.72-0.79)	0.77 (0.73-0.80)	0.75 (0.69-0.84)	0.81 (0.73-0.90)

Abbreviations: AIRR, adjusted incidence rate ratio; MET, metabolic equivalent task; MVPA, moderate to vigorous physical activity; PA, physical activity.

^a^ Model adjusted for age (continuous), sex (reference, male), and wave (reference, baseline).

^b^ Model adjusted for age (continuous), sex (reference, male), wave (reference, baseline), educational level (reference, primary), smoking status (reference, nonsmoker), excess alcohol use (reference, none), and antidepressant medication use (reference, nonuse).

### Analysis of MVPA Doses and Major Depression Status in Overall Sample

Table 2 reports on doses and major depression status. A dose-response relationship between physical activity dose categories and major depression was observed. For the 3 dose categories, compared with low-dose physical activity, high-dose physical activity showed 44% lower odds of major depression (AOR, 0.56; 95% CI, 0.48-0.65). For the 5 dose categories, 600 to less than 1200 MET-min/wk was associated with 44% lower odds of major depression (AOR, 0.56; 95% CI, 0.43-0.73), and 2400 MET-min/wk or more was associated with 49% (AOR, 0.51; 95% CI, 0.43-0.62) lower odds of major depression. Bonferroni-corrected post hoc analysis (Table 2) showed significantly lower odds of major depression for 400 to less than 600 MET-min/wk compared with participants performing 0 MET-min/wk (AOR, 0.57; 95% CI, 0.49-0.66). No association between continuous MET minutes per week and major depression was found.

### MVPA Dose and Major Depression Status Among Older Adults With and Without Chronic Disease

Table 4 presents findings on dose categories and major depression status among older adults with and without chronic disease. For the 3 dose categories, compared with low-dose physical activity, moderate physical activity was associated with 42% lower odds of major depression (AOR, 0.58; 95% CI, 0.43-0.76) for those with chronic disease and, for those without chronic disease, high-dose physical activity was associated with 35% lower odds of major depression (AOR, 0.65; 95% CI, 0.45-0.95). For the 5 dose categories, significantly lower odds of major depression were found beginning at 600 to less than 1200 MET-min/wk among those with disease (AOR, 0.56; 95% CI, 0.42-0.74), with the lowest odds at 2400 MET-min/wk or more (AOR, 0.51; 95% CI, 0.42-0.63). Null findings were materially the same among those without disease after full adjustment. No association between continuous MET minutes per week and major depression was found.

**Table 4.** AORs for Associations of MVPA With Major Depression Status in Those With and Without Chronic Disease

PA dose type (MET∙min∙wk^−1^)	With disease (n = 16 204)		Without disease (n = 3490)	
	AOR (95% CI)^a^	AOR (95% CI)^b^	AOR (95% CI)^a^	AOR (95% CI)^b^
Continuous	1.00 (1.00-1.00)	1.00 (1.00-1.00)	1.00 (1.00-1.00)	1.00 (1.00-1.00)
3 Dose PA				
Low	1 [Reference]	1 [Reference]	1 [Reference]	1 [Reference]
Moderate	0.50 (0.39-0.65)	0.58 (0.43-0.76)	0.68 (0.38-1.21)	0.60 (0.30-1.18)
High	0.52 (0.44-0.61)	0.56 (0.47-0.66)	0.53 (0.38-0.73)	0.65 (0.45-0.95)
5 Dose PA				
0	1 [Reference]	1 [Reference]	1 [Reference]	1 [Reference]
1 to <600	0.74 (0.59-0.91)	0.84 (0.67-1.06)	0.78 (0.47-1.30)	1.03 (0.59-1.82)
600 to <1200	0.47 (0.36-0.61)	0.56 (0.42-0.74)	0.64 (0.36-1.14)	0.60 (0.30-1.22)
1200 to <2400	0.55 (0.44-0.70)	0.60 (0.47-0.77)	0.50 (0.30-0.84)	0.67 (0.38-1.19)
≥2400	0.45 (0.38-0.55)	0.51 (0.42-0.63)	0.49 (0.34-0.72)	0.65 (0.41-1.02)

Abbreviations: AOR, adjusted odds ratio; MET, metabolic equivalent task; MVPA, moderate to vigorous physical activity; PA, physical activity.

^a^ Model adjusted for age (continuous), sex (reference, male), and wave (reference, baseline).

^b^ Model adjusted for age (continuous), sex (reference, male), wave (reference, baseline), educational level (reference, primary), smoking status (reference, nonsmoker), excess alcohol use (reference, none), and antidepressant medication use (reference, nonuse).

### Sensitivity Analysis

Results were materially the same in sensitivity analyses for both depressive symptoms and major depression status. The associations of all variables included in the fully adjusted models with the outcome measures are presented in eTables 1-3 in Supplement 1.

## Discussion

The present findings appear to support the protective influence of MVPA for depressive symptoms and major depression, which is consistent with previous reports and previous work from TILDA.^8,12,13,14,15^ To our knowledge, this is the first longitudinal investigation from a nationally representative cohort of older adults with and without chronic disease to observe that a lower dose of MVPA than that recommended from WHO guidelines for overall health was associated with a reduced risk of depressive symptoms and major depression during a 10-year period. Older adults performing 400 to less than 600 MET-min/wk had a 16% lower rate of depressive symptoms and 43% lower odds of major depression. These findings are consistent with recent meta-analytic data suggesting that salutary mental health benefits among adults can be achieved with physical activity below public health recommendations; specifically, an activity volume equivalent to 2.5 hours per week of brisk walking was associated with a 25% lower risk of depression, and half that activity volume was associated with an 18% lower risk compared with no activity.^12^ Herein, the findings suggest that accumulating as little as 100 minutes per week or 20 minutes per day for 5 days per week of moderate-intensity activity (eg, brisk walking; 4 METs) may be sufficient to significantly lower the risk of depressive symptoms and odds of major depression over time among older adults.

Moreover, the present findings appear to support a dose-response association between MVPA and depression; the magnitude of the reduced risk increased as the dose increased, with 7% lower risk for doses meeting recommendations, 16% lower risk for doses exceeding recommendations, and 23% lower risk for the highest doses. The highest dose category (ie, ≥2400 MET-min/wk) was associated with the largest magnitude of odds reductions for both depressive symptoms (23%) and major depression (49%). The magnitude of odds reductions for major depression are consistent with and/or exceed the odds for reductions following physical activity exposure from recent meta-analytic evidence,^7^ which suggested larger odds reductions over time as the dose increased, and previous prospective cohort findings from TILDA.^8^ Although engaging in such a high dose of physical activity has well-established salutary benefits among older adults,^16,17^ it is also plausible that older adults who engage in this high level also engage in other healthy beneficial behaviors. These can include healthier dietary patterns^18^ and higher social engagement levels,^19^ which could also plausibly reduce the risk of experiencing depressive symptoms.^20,21^

The present findings also suggest that minimally sufficient activity doses for depressive symptoms and major depression vary based on chronic disease status. For depressive symptoms, older adults with disease showed a significantly reduced risk (7%) at the WHO guideline threshold of 600 MET-min/wk, although the greatest decreases occurred with increasing physical activity dose, with a similar outcome observed for major depression. These findings are consistent with previous evidence indicating that adults with chronic illness may require higher physical activity doses for greater benefits^22,23^ and previous meta-analytic evidence that suggested significantly larger antidepressant effects of exercise training among adults with chronic illness who were meeting WHO guidelines for physical activity.^10^ The present findings suggesting that lower doses of physical activity may be beneficial for depression are also consistent with previous evidence that supported the benefits of lower doses of and small increases in MVPA for all-cause mortality and chronic disease. For example, previous findings suggest that approximately 110 000 deaths per year could be prevented if adults aged 40 to 85 years increased MVPA by even 10 minutes per day.^24^

In individuals without disease, a significantly reduced risk of depressive symptoms was found only at the highest physical activity doses of 2400 MET-min/wk or more. This suggests that older adults without chronic disease may need a higher physical activity dose to protect against depressive symptoms. This was not surprising given that individuals without chronic conditions could already be engaged in salutary lifestyle practices,^17,18,19^ resulting in less likelihood of experiencing increased depressive symptoms and major depression compared with those with a chronic disease, and thus may require greater physical activity doses to elicit further protection.

As anticipated based on logical, theoretical, and/or prior empirical association with physical activity, depressive symptoms, and/or their associations, eTable 1 in Supplement 1 reports that female sex, lower educational level, smoking, alcohol use, and antidepressant use were significant covariates in the models for depressive symptoms and major depression. These findings are consistent with previous research from TILDA into longitudinal associations between lifestyle factors (including physical activity and smoking) with depression, anxiety, and generalized anxiety disorder that showed greater odds of impaired mental health and lower physical activity particularly among females, smokers, and those with excessive alcohol use.^8,25,26,27^ Although beyond the scope of the current investigation, these factors, along with immunoserotonergic interactions, neuroendocrine factors, neurovisceral integration (or heart-brain crosstalk), neurotrophic peptides, and the interplay of biopsychosocial factors,^28,29^ warrant additional investigation in an effort to further explain the physical activity–depression association.

### Public Health Implications

These findings suggest that physical activity at lower doses than WHO recommendations for overall health may offer protection against depressive symptoms and major depression among older adults. We do not advocate for reduced activity levels in any population, but these findings suggest that even doses lower than recommended may protect mental health over time. It may be useful for public health agencies and clinicians to implement evidence-based strategies to promote these potentially more acceptable and achievable doses of physical activity to reduce the adverse consequences of elevated depressive symptoms, including reducing the associated burden of conditions for which depression is a risk factor, such as cardiovascular disease, diabetes, chronic pain, and anxiety disorders. Furthermore, minimally sufficient doses to protect against depressive symptoms and major depression may vary based on chronic disease status, which needs to be considered when advocating physical activity for prevention and management.

### Strengths and Limitations

Strengths of the current study include (1) a nationally representative cohort of older adults for whom detailed demographic, health, lifestyle, exposure, and outcome variable data were available at 5 repeated measures over a decade; (2) analyses of older adults with and without chronic disease; (3) investigation of more precise, particularly lower, MVPA exposures to better estimate minimal doses; and (4) adjustment for several plausible confounders in all groups of interest.

The study was not without limitations that warrant consideration when interpreting findings. Moderate to vigorous physical activity and depressive symptoms, but not major depression, from the Composite International Diagnostic Interview were self-reported, risking overreporting or underreporting and potential social desirability biases, and depression was not controlled for family history. This analysis also did not consider chronic pain or sleep information, which may influence the association. Despite the temporal sequence of the prospective design, the measure of the associations found, the established biological plausibility of the physical activity and depression association, and independence from important confounders, the cohort design does not allow causality to be inferred from these findings. Data on race and ethnicity were not obtained; to enhance generalizability, these findings should be replicated in more diverse samples. Future studies also should use device-measured physical activity to corroborate self-reported activity.

## Conclusions

The findings of this cohort study suggest that physical activity doses lower (ie, 400 to <600 MET-min/wk) than doses recommended in guidelines for overall health (ie, ≥600 MET-min/wk) may protect against depressive symptoms and major depression among older adults. However, greater physical activity doses were associated with larger risk reductions and reduced AORs. It may be useful for public health interventions to investigate acceptance and achievability of minimal physical activity thresholds among older adults with and without chronic disease to reduce the risk of depression.

## Notes

Supplement 1. **eFigure.** The Major Depression Status of TILDA Participants (n = 4016) Across Data Collection Waves 1-5 **eTable 1.** Adjusted Incident Rate Ratios for Associations of All Included Variables With Depressive Symptoms and Adjusted Odds Ratios for Associations With Major Depression Status **eTable 2.** Adjusted Incident Rate Ratios for Associations of All Included Variables With Depressive Symptoms in Those With and Without Chronic Disease **eTable 3.** Adjusted Odd Ratios for All Included Variables With Major Depression Status in Those With and Without Chronic Disease

Supplement 2.
Data Sharing Statement
