# Supplementary material for: Physical Activity Dose and Depression in a Cohort of Older Adults in The Irish Longitudinal Study on Ageing
Source: JAMA Netw Open. 2023 Jul 10;6(7):e2322489. doi: 10.1001/jamanetworkopen.2023.22489 (PMC10334250; doi:10.1001/jamanetworkopen.2023.22489)
Supplement: Supplement 1. — eFigure. The Major Depression Status of TILDA Participants (n = 4016) Across Data Collection Waves 1-5 eTable 1. Adjusted Incident Rate Ratios for Associations of All Included Variables With Depressive Symptoms and Adjusted Odds Ratios for Associations With Major Depression Status eTable 2. Adjusted Incident Rate Ratios for Associations of All Included Variables With Depressive Symptoms in Those With and Without Chronic Disease eTable 3. Adjusted Odd Ratios for All Included Variables With Major Depression Status in Those With and Without Chronic Disease [file jamanetwopen-e2322489-s001.pdf]

Supplementary Online Content

Laird E, Rasmussen CL, Kenny RA, Herring MP. Physical activity dose and depression in a cohort of older adults in The Irish Longitudinal Study on Ageing. *JAMA Netw Open*. 2023;6(7):e2322489. doi:10.1001/jamanetworkopen.2023.22489

eFigure. The Major Depression Status of TILDA Participants (n = 4016) Across Data Collection Waves 1-5

eTable 1. Adjusted Incident Rate Ratios for Associations of All Included Variables With Depressive Symptoms and Adjusted Odds Ratios for Associations With Major Depression Status

eTable 2. Adjusted Incident Rate Ratios for Associations of All Included Variables With Depressive Symptoms in Those With and Without Chronic Disease

eTable 3. Adjusted Odd Ratios for All Included Variables With Major Depression Status in Those With and Without Chronic Disease

This supplementary material has been provided by the authors to give readers additional information about their work.

© 2023 Laird E et al. *JAMA Network Open.*

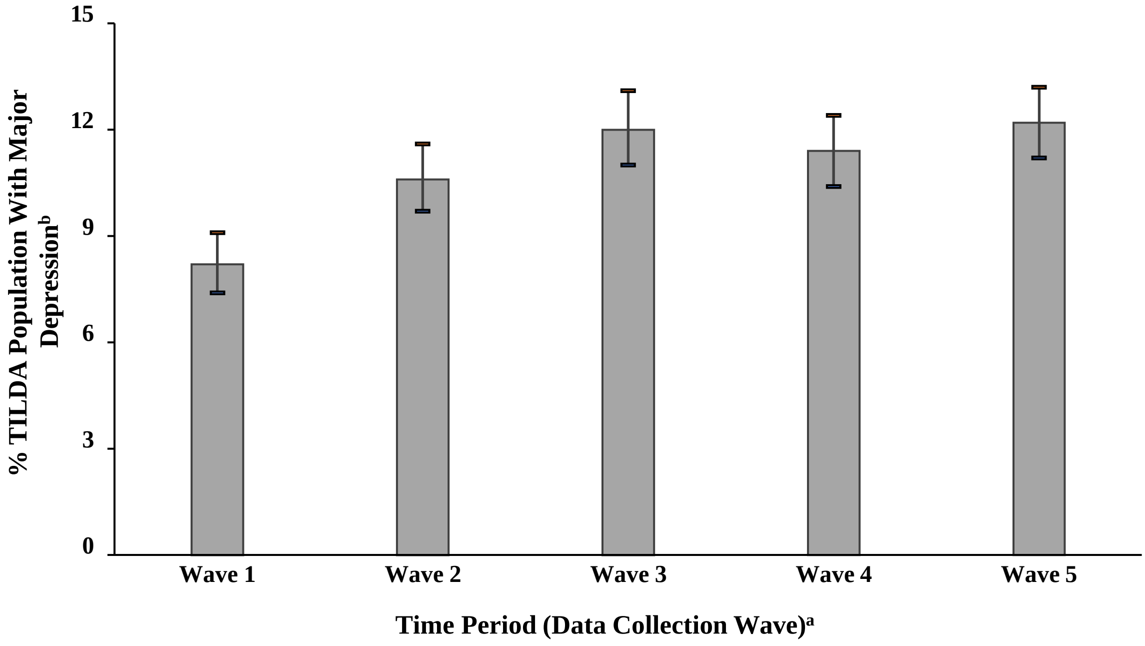

eFigure. The Major Depression Status Of TILDA Participants (n = 4016) Across Data Collection Waves 1-5.

[a]Values at each Wave indicate mean percentage (%) with 95% Confidence Intervals
[b]At all Waves, depressive symptoms were measured using the short-form of Centre for Epidemiological Studies Depression (CES-D), with a score of ≥9 used to define clinically significant depression status. From Waves 2-5, the Composite International Diagnostic Interview quantified diagnosis of a Major Depressive Episode during the past 12 months; Major Depression was classified as either a CES-D≥9 (at any Wave) and/or a diagnosis of Major Depressive Episode (Waves 2-5).

© 2023 Laird E et al. *JAMA Network Open.*

eTable 1. Adjusted Incident Rate Ratios For Associations Of All Included Variables With Depressive Symptoms And Adjusted Odds Ratios For Associations With Major Depression Status

	Depressive symptoms			Major Depression status		
	IRR (95% CI)			OR (95% CI)		
Variables	Continuous model	3 dose PA model	5 dose PA model	Continuous model	3 dose PA model	5 dose PA model
Age, y	1.00 (1.00-1.00)	1.00 (1.00-1.00)	1.00 (1.00-1.00)	0.98 (0.96-0.99)	0.97 (0.96-0.99)	0.97 (0.96-0.98)
Sex[a]						
Female	1.31 (1.24-1.39)	1.31 (1.24-1.39)	1.30 (1.23-1.38)	1.90 (1.57-2.30)	1.87 (1.55-2.26)	1.85 (1.53-2.24)
Education level[a]						
Secondary	0.88 (0.81-0.94)	0.88 (0.82-0.95)	0.88 (0.82-0.95)	0.73 (0.57-0.93)	0.74 (0.58-0.94)	0.74 (0.58-0.94)
Tertiary	0.86 (0.80-0.92)	0.87 (0.81-0.94)	0.87 (0.81-0.94)	0.59 (0.46-0.75)	0.61 (0.47-0.78)	0.61 (0.48-0.78)
Lifestyle factors[a]						
Current smoker	1.12 (1.04-1.20)	1.11 (1.04-1.19)	1.11 (1.04-1.19)	1.97 (1.56-2.50)	1.97 (1.56-2.49)	1.96 (1.55-2.48)
Alcohol issue	1.19 (1.13-1.26)	1.19 (1.13-1.26)	1.20 (1.13-1.27)	1.40 (1.12-1.74)	1.41 (1.13-1.76)	1.41 (1.13-1.76)
Anti-depressant use	1.34 (1.25-1.43)	1.34 (1.26-1.43)	1.34 (1.26-1.43)	5.38 (4.31-6.73)	5.37 (4.30-6.71)	5.36 (4.29-6.70)
Time period[a]						
Wave 2	0.92 (0.88-0.96)	0.92 (0.88-0.96)	0.92 (0.88-0.97)	1.45 (1.19-1.77)	1.48 (1.21-1.80)	1.49 (1.22-1.81)
Wave 3	1.15 (1.09-1.20)	1.14 (1.09-1.19)	1.14 (1.09-1.19)	1.75 (1.43-2.13)	1.72 (1.41-2.11)	1.72 (1.41-2.11)
Wave 4	1.15 (1.09-1.20)	1.14 (1.09-1.19)	1.14 (1.09-1.20)	1.81 (1.47-2.23)	1.80 (1.47-2.22)	1.81 (1.47-2.23)
Wave 5	1.19 (1.13-1.25)	1.18 (1.12-1.24)	1.18 (1.12-1.24)	2.03 (1.63-2.53)	2.03 (1.63-2.52)	2.03 (1.63-2.53)
PA dose (MET.min.week^{-1})						
Continuous, min/wk	1.00 (1.00-1.00)	-	-	1.00 (1.00-1.00)	-	-
3 dose PA						
Low PA	-	[Reference]	-	-	[Reference]	-
Moderate PA	-	0.95 (0.90-1.00)	-	-	0.58 (0.45-0.75)	-
High PA	-	0.81 (0.78-0.84)	-	-	0.56 (0.48-0.65)	-
5 dose PA						
0	-	-	[Reference]	-	-	[Reference]
1-<600	-	-	0.94 (0.89-0.99)	-	-	0.86 (0.69-1.06)
600-<1,200	-	-	0.93 (0.88-0.99)	-	-	0.56 (0.43-0.73)
1200-<2400	-	-	0.84 (0.80-0.89)	-	-	0.59 (0.47-0.74)

© 2023 Laird E et al. *JAMA Network Open.*

≥2,400	-	-	0.77 (0.74-0.80)	-	-	0.51 (0.43-0.62)

Abbreviations: PA, physical activity; IRR, incidence rate ratio; OR, Odds ratio; CI, confidence interval.

[a]Reference categories: age [continuous], sex [reference, male], wave [reference, baseline], education [reference, primary], smoking status [reference, non-smoker], alcohol issue [reference, none], anti-depressant medication use [reference, non-user].

© 2023 Laird E et al. *JAMA Network Open.*

eTable 2. Adjusted Incident Rate Ratios For Associations Of All Included Variables With Depressive Symptoms In Those With And Without Chronic Disease

	With chronic Disease (MET.min.week^{-1}) IRR (95% CI)			Without chronic Disease (MET.min.week^{-1}) IRR (95% CI)		
Variables	Continuous model	3 dose PA model	5 dose PA model	Continuous model	3 dose PA model	5 dose PA model
Age, y	1.00 (0.99-1.00)	1.00 (0.99-1.00)	1.00 (0.99-1.00)	0.99 (0.98-1.00)	0.99 (0.99-1.00)	0.99 (0.98-1.00)
Sex*						
Female	1.29 (1.22-1.37)	1.29 (1.22-1.37)	1.29 (1.21-1.36)	1.25 (1.11-1.41)	1.26 (1.12-1.43)	1.26 (1.11-1.42)
Education level*						
Secondary	0.89 (0.82-0.96)	0.89 (0.83-0.97)	0.90 (0.83-0.97)	0.96 (0.80-1.15)	0.97 (0.81-1.15)	0.96 (0.80-1.14)
Tertiary	0.84 (0.78-0.91)	0.86 (0.79-0.93)	0.86 (0.79-0.93)	0.99 (0.83-1.19)	1.00 (0.84-1.20)	1.00 (0.83-1.19)
Lifestyle factors*						
Current smoker	1.14 (1.06-1.23)	1.14 (1.06-1.23)	1.14 (1.06-1.23)	1.11 (0.95-1.29)	1.10 (0.94-1.29)	1.09 (0.93-1.27)
Alcohol issue	1.19 (1.12-1.26)	1.19 (1.12-1.27)	1.19 (1.12-1.27)	1.24 (1.08-1.43)	1.25 (1.09-1.44)	1.24 (1.08-1.43)
Anti-depressant use	1.31 (1.23-1.40)	1.31 (1.23-1.40)	1.32 (1.23-1.41)	1.76 (1.43-2.15)	1.75 (1.43-2.15)	1.75 (1.43-2.15)
Time period*						
Wave 2	0.92 (0.87-0.97)	0.92 (0.87-0.97)	0.92 (0.88-0.97)	0.97 (0.87-0.97)	0.96 (0.85-1.08)	0.96 (0.86-1.08)
Wave 3	1.17 (1.11-1.22)	1.16 (1.10-1.22)	1.16 (1.10-1.22)	1.17 (1.11-1.22)	1.14 (1.01-1.29)	1.14 (1.01-1.29)
Wave 4	1.16 (1.10-1.22)	1.15 (1.10-1.21)	1.16 (1.10-1.22)	1.16 (1.10-1.22)	1.16 (1.02-1.32)	1.16 (1.02-1.33)
Wave 5	1.21 (1.14-1.28)	1.20 (1.13-1.27)	1.20 (1.14-1.27)	1.21 (1.14-1.28)	1.21 (1.05-1.39)	1.21 (1.05-1.40)
PA dose (MET.min.week^{-1})						
Continuous, min/wk	1.00 (1.00-1.00)	-	-	1.00 (1.00-1.00)	-	-
3 dose PA		-	-		-	-
Low PA	-	[Reference]		-	[Reference]	
Moderate PA	-	0.93 (0.88; 0.99)	-	-	1.04 (0.89-1.21)	-
High PA	-	0.79 (0.76; 0.83)	-	-	0.90 (0.82-0.99)	-
5 dose PA						
0	-	-	[Reference]	-	-	[Reference]
1-<600	-	-	0.95 (0.90-1.01)	-	-	0.86 (0.74-0.99)
600-<1,200	-	-	0.92 (0.87-0.98)	-	-	1.00 (0.85-1.16)
1200-<2400	-	-	0.82 (0.78-0.87)	-	-	0.99 (0.87-1.13)
≥2,400	-	-	0.77 (0.73-0.80)	-	-	0.80 (0.72-0.90)

© 2023 Laird E et al. *JAMA Network Open.*

Abbreviations: PA, physical activity; IRR, incidence rate ratio; CI, confidence interval.
[*]Reference categories: age [continuous], sex [reference, male], wave [reference, baseline], education [reference, primary], smoking status [reference, non-smoker], alcohol issue [reference, none], anti-depressant medication use [reference, non-user].

© 2023 Laird E et al. *JAMA Network Open.*

eTable 3. Adjusted Odd Ratios For All Included Variables With Major Depression Status In Those With And Without Chronic Disease

	OR (95% CI)			OR (95% CI)		
Variables	Continuous model	3 dose PA model	5 dose PA model	Continuous model	3 dose PA model	5 dose PA model
Age, y	0.97 (0.96-0.98)	0.97 (0.96-0.98)	0.97 (0.96-0.98)	0.94 (0.91-0.97)	0.94 (0.91-0.97)	0.94 (0.91-0.97)
Sex*						
Female	1.78 (1.45-2.18)	1.76 (1.44-2.15)	1.74 (1.43-2.13)	1.93 (1.24-3.00)	1.83 (1.17-2.84)	1.83 (1.18-2.85)
Education level*						
Secondary	0.72 (0.56-0.93)	0.73 (0.57-0.94)	0.74 (0.57-0.95)	1.11 (0.57-2.16)	1.08 (0.56-2.09)	1.08 (0.56-2.09)
Tertiary	0.60 (0.46-0.78)	0.62 (0.48-0.81)	0.62 (0.48-0.81)	0.71 (0.36-1.42)	0.71 (0.36-1.42)	0.71 (0.36-1.43)
Lifestyle factors*						
Current smoker	2.12 (1.64-2.73)	2.12 (1.64-2.73)	2.11 (1.63-2.72)	1.46 (0.85-2.51)	1.43 (0.83-2.46)	1.43 (0.83-2.47)
Alcohol issue	1.26 (1.00-1.60)	1.27 (1.00-1.62)	1.28 (1.01-1.62)	2.19 (1.31-3.66)	2.22 (1.32-3.71)	2.22 (1.32-3.72)
Anti-depressant use	5.12 (4.05-6.48)	5.11 (4.04-6.45)	5.10 (4.03-6.45)	9.34 (4.74-18.40)	9.37 (4.76-18.43)	9.37 (4.76-18.45)
Time period*						
Wave 2	1.43 (1.15-1.77)	1.45 (1.17-1.80)	1.46 (1.17-1.81)	1.92 (1.12-3.28)	1.97 (1.15-3.38)	1.97 (1.14-3.38)
Wave 3	1.75 (1.41-2.17)	1.72 (1.39-2.14)	1.72 (1.39-2.14)	2.37 (1.34-4.17)	2.35 (1.33-4.15)	2.35 (1.33-4.15)
Wave 4	1.82 (1.46-2.28)	1.80 (1.44-2.25)	1.81 (1.45-2.26)	2.41 (1.30-4.46)	2.46 (1.32-4.56)	2.46 (1.32-4.57)
Wave 5	2.02 (1.59-2.55)	2.01 (1.58-2.54)	2.01 (1.59-2.55)	3.16 (1.63-6.11)	3.22 (1.66-6.24)	3.22 (1.66-6.24)
PA dose (MET.min.week^{-1})						
Continuous, min/wk	1.00 (1.00-1.00)	-	-	1.00 (1.00-1.00)	-	-
3 dose PA		-	-		-	-
Low PA	-	[Reference]		-	[Reference]	
Moderate PA	-	0.57 (0.43-0.75)	-	-	0.62 (0.31-1.24)	-
High PA	-	0.57 (0.49-0.68)	-	-	0.57 (0.49-0.68)	-
5 dose PA						
0	-	-	[Reference]	-	-	[Reference]
1-<600	-	-	0.84 (0.67-1.06)	-	-	1.00 (0.56-1.80)
600-<1,200	-	-	0.55 (0.42-0.73)	-	-	0.62 (0.30-1.26)
1200-<2400	-	-	0.62 (0.49-0.80)	-	-	0.54 (0.29-1.00)
≥2,400	-	-	0.52 (0.43-0.63)	-	-	0.57 (0.36-0.91)

© 2023 Laird E et al. *JAMA Network Open.*

Abbreviations: PA, physical activity; OR, odds ratio; CI, confidence interval.
[*]Reference categories: age [continuous], sex [reference, male], wave [reference, baseline], education [reference, primary], smoking status [reference, non-smoker], alcohol issue [reference, none], anti-depressant medication use [reference, non-user]

© 2023 Laird E et al. *JAMA Network Open.*
