# Supplementary material for: Physical Activity Dose and Depression in a Cohort of Older Adults in The Irish Longitudinal Study on Ageing
Source: JAMA Netw Open. 2023 Jul 10;6(7):e2322489. doi: 10.1001/jamanetworkopen.2023.22489 (PMC10334250; doi:10.1001/jamanetworkopen.2023.22489)
Supplement: Supplement 2. — Data Sharing Statement [file jamanetwopen-e2322489-s002.pdf]

Data Sharing Statement

Laird. Physical Activity Dose and Depression in a Cohort of Older Adults in The Irish Longitudinal Study on Ageing. *JAMA Netw Open*. Published July 10, 2023. doi:10.1001/jamanetworkopen.2023.22489

Data
Data available: No

Additional Information
Explanation for why data not available: Code for the analysis will ne made available. The actual data is protected and researchers can apply for access
